# Reply to Brunner, M. Comment on “Värendh et al. A Cost-Effectiveness Analysis of the Sentio Bone Conduction Hearing Implant System in the Australian Healthcare Setting. *J. Mark. Access Health Policy* 2026, *14*, 8”

**DOI:** 10.3390/jmahp14040060

**Published:** 2026-10-05

**Authors:** Magnus Värendh, Ida Haggren, Helén Lagerkvist, Maria Åberg Håkansson, Jonas Hjelmgren

**Affiliations:** 1 IHE—The Swedish Institute for Health Economics, 223 61 Lund, Sweden; 2 Department of Economics, Lund University, 220 07 Lund, Sweden; 3 Oticon Medical AB, 436 32 Askim, Sweden

**Overall** **Comments**

We read with interest the cost-effectiveness analysis by Värendh et al. comparing the Sentio and Osia bone conduction hearing implant (BCHI) systems in the Australian healthcare setting. While we appreciate efforts to evaluate the recently introduced Sentio system, substantial methodological flaws and unvalidated assumptions, including inconsistency with Sentio’s instructions for use, systematically bias results in favor of Sentio, undermining the authors’ conclusion of economic dominance over Osia. We highlight four critical concerns that compromise the study’s validity for clinical and health technology assessment (HTA) decision making.

*Authors’* *response:*

We thank the author for his careful reading and welcome the opportunity to respond. Several of the concerns raised in the letter [1] are directly addressed in the manuscript’s sensitivity and scenario analyses and further discussed in the Discussion Section. To the greatest extent possible, the analysis adheres to standard health-economic modeling methodology (Caro et al. 2012 [2]; Briggs et al. 2006 [3]; Husereau et al. 2022 [4]). The model itself is an adaptation of the Markov cost–utility framework published by Brunner et al. 2024 [5] in this journal, with external validation against Brunner’s published Osia–Baha Attract results yielding ICERs within 0.1% of the original (Table A5, Appendix C, in Värendh et al. (2026) [6]). We respond to each of the four concerns in turn below.

**Comment 1.** **Disparate Clinical Evidence Standards**

A fundamental flaw is the profound disparity in clinical evidence quality between the two devices. The authors acknowledge that “Sentio is a recently introduced device” with “limited long-term data” yet proceed as though evidence bases were equivalent. This violates HTA principles requiring comparable evidence quality across comparators. For complication rates—a key driver of both quality-adjusted life years (QALYs) and costs—the Osia evidence derives from a systematic review and meta-regression by Caversaccio et al. synthesizing 12 studies involving patients in whom an Osia system had been implanted. In contrast, the Sentio evidence relies primarily on a single 51-patient study by Hol et al. with a six-month follow-up, supplemented by proxy data obtained from predecessor devices.

Critically, the Hol study reported zero explantations at six months. This does not constitute evidence of zero risk; instead, it reflects insufficient statistical power to detect rare events in a small sample over a short duration. The authors could have avoided this asymmetry by using the comparable Osia study by Briggs et al. with a six-month follow-up, which also showed zero explantations. Instead, they applied disparate evidence standards that inherently favor Sentio.

*Authors’* *response:*

The asymmetry in the evidence base between Sentio and the more established comparators reflects the current state of the published BCHI literature, and we acknowledge it openly in the manuscript. Combining heterogeneous sources of evidence—pooled meta-analytic data for established devices alongside efficacy and early-effectiveness data for newer ones—is standard practice in health-economic modeling under conditions of asymmetric evidence and is the explicit recommendation of the ISPOR-SMDM Modelling Good Research Practices Task Force (Caro et al. 2012 [2]; Briggs et al. 2012 [7]).

For Osia, Ponto, and Baha Attract, base-case event probabilities were drawn from the follow-up-adjusted meta-regression by Caversaccio et al. 2025 [8], the most comprehensive pooled safety evidence currently available. For Sentio, base-case inputs were drawn from the five-year follow-up of the predecessor BCI (Reinfeldt et al. 2022 [9]), with the six-month Sentio data from Hol et al. 2026 [10] reserved for scenario analysis. The letter’s characterization of our Sentio evidence as “primarily a single 51-patient study by Hol et al.” therefore inverts the actual hierarchy used in the model. Uncertainty was further handled through three complementary mechanisms: (i) a 10,000-iteration probabilistic sensitivity analysis varying all parameters with available distributions; (ii) deterministic scenario analyses substituting alternative evidence sources for Sentio, including the proxy the letter recommends; and (iii) structured clinical expert validation of model assumptions.

The most directly relevant scenario applies the pooled aBCIem rates from Caversaccio et al. 2025 [8], the exact benchmark the letter recommends, to Sentio. In this scenario, 92% of the modeled QALY gain is removed (Table A4 in Appendix B, in Värendh et al. (2026) [6]), yet Sentio still dominates Osia. A separate scenario applying the short-term Sentio data from Hol et al. produces a 3.9% QALY decrease, with Sentio still dominant. A matched short-follow-up scenario using Briggs et al. 2022 [11] and Hol et al. 2026 [10], both reporting zero explantations at six months, would similarly not change the conclusion. The cost-effectiveness conclusion is therefore not contingent on the specific Sentio evidence source used.

The manuscript states throughout that the Sentio evidence base is limited and that direct long-term observational data on reimplantation, complication rates, and patient-reported outcomes for Sentio remain a priority for future research. The distinction between facts (published data, applied with their reported uncertainty) and assumptions (parameter choices made where data are absent or limited, tested through sensitivity analysis) is observed throughout—and is the principal reason such models exist.

**Comment 2.** **Unsupported Equal-Utility Assumption**

The authors assume identical patient utility (0.76 for the On-BCHI state) for both devices despite having no Sentio-specific utility data. While health-related quality-of-life utility data were collected in key Osia clinical studies using the HUI-3 instrument, no such data exist for Sentio patients. The equal-utility assumption rests entirely on “clinical expertise validation” that involved four unidentified international experts and was conducted through an undisclosed “semi-structured” process. This validation approach lacks transparency regarding expert identities, potential conflicts of interest, questions posed, evidence reviewed, access to actual Sentio patient-reported outcomes, and validation criteria used. From a clinical and HTA perspective, assuming equal effectiveness without supporting data contradicts evidence-based decision-making principles. More importantly, this assumption fundamentally undermines the dominance claim. The authors explicitly acknowledge that “incremental QALY differences in the model are driven by modeled event pathways and cost offsets rather than demonstrated differences in clinical effectiveness”. The claimed QALY difference over 15 years (approximately 0.007 QALYs annually, equivalent to 2.7 days of perfect health per year) derives entirely from differences in modeled complication rates, not from superior patient-reported outcomes, better hearing performance, or improved quality of life. Sentio demonstrates no quality-of-life advantage over Osia according to the model’s own logic.

*Authors’* *response:*

The absence of Sentio-specific utility data is an important limitation, and we acknowledge it prominently in the manuscript. We did not claim that Sentio offers a quality-of-life advantage over Osia, nor does the model require this.

While it remains true that no HUI3 data exist for Sentio patients, validated hearing-related quality-of-life questionnaires for Sentio are reported in Hol et al. 2026 [10]. The equal-utility assumption does not rest entirely on clinical expert validation; it is supported by the literature and reported performance outcomes described in Section 2.5.6 in Värendh et al. (2026) [6], which states that *“clinically relevant differences are more linked to the type of hearing loss than the type of BCHIs”* (Gutierrez et al. 2024 [12]), and which sets out overlapping GBI scores across Sentio, Ponto, and pooled transcutaneous BCHIs; equivalent functional gain ranges for Sentio and Osia; and similar SSQ patient-reported outcomes between Sentio and Osia. The full mapping of patient-reported and audiological outcomes across systems is summarized in Table 1 below:

The clinical relevance of this convergent evidence, i.e., whether it supports treating the BCHI systems Osia, Ponto, and Sentio as providing comparable quality-of-life improvements such that the same utility weights can be applied across them, was specifically posed to the clinical experts as part of the validation. The use of structured expert input in the absence of direct comparative utility data is a recognized practice in health-economic modeling and is the subject of an ISPOR Good Practices report on structured expert elicitation (Soares et al. 2024 [14]). The validation included an introduction to the project and model, followed by a structured interview covering (i) whether the model adequately reflects clinical pathways and decision points; (ii) whether the assumption of one AE-driven reimplantation, with a second resulting in transition to the off-BCHI state, reflects clinical practice; (iii) whether the BCHI systems Osia, Ponto, and Sentio provide comparable quality-of-life improvements such that the same utility weights can be applied across them; (iv) whether planned reimplantation after 10 years to compensate for implant wear and/or hearing decline is realistic; and (v) whether extending the planned-reimplantation interval to 15 years is realistic for a more powerful implant. The four clinical experts have between >15 and >25 years of experience using BCHIs across systems and models, together representing more than 75 years of relevant insights, with two of the experts being appointed professors. One of the four experts opted for anonymity; out of respect for that preference, we did not disclose the full names of the others. The reporting of the validation, including the anonymous treatment of expert input, was reviewed and accepted during journal peer review.

Robustness to the equal-utility assumption was tested directly. In the univariate sensitivity analysis, the *on-BCHI* utility was identified as the single most influential parameter, with *off-BCHI* utility ranked third (Section 3.2.1 in Värendh et al. (2026) [6]). Scenario analyses perturbing Sentio utility by ±5% and ±10% relative to Osia (Table A4 in Värendh et al. (2026) [6]) show Sentio remaining economically favorable across the range: under positive perturbations, Sentio remains dominant; under negative perturbations, Sentio shifts to “less costly, less effective” but never becomes economically unfavorable.

The manuscript is explicit about what drives the modeled QALY difference. We did not claim clinical superiority:

*“Health state utilities specific to Sentio were unavailable… The absence of Sentio-specific health state utility data increases uncertainty in the analysis, with potential impact on the ICER, and is therefore an important limitation.”*

(Section 4 in Värendh et al. (2026) [6])

*“Incremental QALY differences in the model are driven by modelled event pathways and cost offsets rather than demonstrated differences in clinical effectiveness.”*

(Section 4 in Värendh et al. (2026) [6])

*“The magnitude of the incremental QALY gain was modest and likely within the measurement uncertainty of generic utility instruments, such as HUI, and therefore should not be interpreted as indicating clinically meaningful superiority.”*

(Section 4, in Värendh et al. (2026) [6])

*“Dominance is not a fixed property of the intervention, but rather a function of specific modelling assumptions, particularly those related to utility inputs.”*

(Section 4, in Värendh et al. (2026) [6])

The annualized QALY figure the letter cites (~0.007 QALYs/year) is consistent with our characterization of the gain throughout the manuscript as “modest” and “small”. Although there are no superior patient-reported outcomes or instances of better hearing performance with Sentio, more time spent on-treatment ultimately impacts quality of life and treatment benefits—which is what the modeled event-pathway difference captures.

**Comment 3.** **Inappropriate Handling of Zero-Event Data**

A fundamental statistical flaw concerns the zero-event explantation rates for Sentio. The model assumes there is zero probability of explantation based on observing no events in 51 patients followed for only six months. In clinical research, when zero events occur in small samples, an absence of evidence is not evidence of an absence. This assumption is particularly problematic given its crucial role in driving the QALY advantage. Explantation events transition patients permanently to the Off-BCHI health state with lower quality of life equivalent to pre-operative, unaided hearing. Since the authors assume equal utility with either functioning device, the QALY benefit attributed to Sentio stems almost exclusively from assuming there are zero explantations over 15 years. This places extraordinary weight on an assumption derived from inadequate evidence, transforming a small sample observation into the primary driver of cost-effectiveness. In the scenario analysis where the explantation rate is set to a more realistic proxy input derived from another electromagnetic active BCHI as reported by Caversaccio, 92% of the estimated QALY gain is lost, demonstrating the extreme sensitivity to this parameter. Compounding this error, zero-event probabilities were treated as fixed in the probabilistic sensitivity analysis, excluding the main driver of utility differences from this important uncertainty assessment. Clinically, this approach fails to acknowledge the real-world uncertainty surrounding long-term Sentio performance.

*Authors’* *response:*

We acknowledge that an absence of evidence is not evidence of an absence, and we treated the Sentio zero-event values accordingly. Section 2.5.5 in Värendh et al. (2026) [6] describes these explicitly as *“empirical zeros based on the available evidence from Reinfeldt* et al. *(predecessor), rather than structural assumptions of no risk. Given the limited sample size and follow-up, these zeros may reflect sparse data rather than the absence of events.”*

The main reason for the empirical zeros is the current evidence base for Sentio: both the most recent publication (Hol et al. 2026 [10], six-month follow-up) and the predecessor study (Reinfeldt et al. 2022 [9], five-year follow-up) registered none of the event types modeled. This could reflect limited follow-up time and sample size, or it could reflect a genuinely lower event rate. To handle this uncertainty, the zero-event parameters were held fixed in the PSA, and their effects were explored separately through scenario analysis using external proxy rates—an approach that makes the impact of substituting non-zero rates directly visible rather than hidden inside a distribution and is standard practice in health-economic modeling under sparse data (Briggs et al. 2012 [7]).

The most informative scenario applies the pooled aBCIem rates from Caversaccio et al. 2025 [8], the exact proxy the letter recommends, to Sentio. The result is reported transparently in Table A4 in Appendix B: an incremental cost of −6721 AUD (−4.7% versus the base case) and an incremental QALY of 0.009 (–92.0% versus the base case), with Sentio still dominant over Osia. A second scenario applying the short-term Sentio data from Hol et al. 2025 [10] reduces the QALY gain by 3.9%, with Sentio still dominant. These results are summarized in Section 3.2.1: *“In both cases, Sentio remained dominant compared with Osia”*, and reinforced in the discussion: *“these scenarios… did not alter the overall economic interpretation. This indicates that the conclusions are not driven by assumptions of zero event risk, but primarily by differences in long-term cost structure.”*

The cost-effectiveness conclusion is therefore not contingent on the Sentio zero-event values: it is robust to substituting them with the precise proxy the letter recommends.

**Comment 4.** **Unvalidated Device Longevity Assumptions**

The model assumes Sentio patients undergo reimplantation after 15 years while Osia patients require reimplantation after 10 years. With only a six-month follow-up, projecting Sentio longevity to 15 years represents extrapolation far beyond any reasonable evidence base. No clinical evidence demonstrates that patients actually experience these differential reimplantation timelines in real-world practice. Of particular note, the assumption for Sentio is inconsistent with the instructions for use, which explicitly state that “The lifetime for the Sentio Ti Implant is ten years”. Reimplantation decisions involve multiple factors, one of which is progressive-hearing-loss severity, and the decision to explant requires clinical assessment and surgeon recommendations. Notably, the fitting range of Sentio’s electromagnetic transducer is limited to 45 dB HL, which can be compared with 55 dB HL for Osia’s piezoelectric transducer. As presented by Breitholtz et al., the wider fitting range of Osia suggests it may compensate for age-related progression of hearing loss longer than Sentio, contradicting the authors’ longevity assumptions. These unsubstantiated device longevity assumptions critically impact the cost-effectiveness conclusions. The modeled cost differential favoring Sentio ($4353) from delayed reimplantation accounts for approximately 62% of the total cost difference between devices ($7050).

*Authors’* *response:*

Although the terms are often used interchangeably in non-technical discussions, *product lifetime* and *expected lifetime* describe different concepts. The product lifetime reflects the *validated* operational life of a device according to industry practices and the *minimum* expected lifetime. It is evident that real-world lifetime data is not available when introducing a device. Additional technical assessments can be performed to explore device aging, but the true lifetime can only be determined from clinical use. To date, the best indication for Sentio lifetime comes from the patients implanted from 2012 and onwards with the BCI implant (Eeg-Olofsson et al. 2014 [15]), who are still using their devices >13 years later. The first model of Osia was introduced in 2019, and we would assume that the most relevant data from actual clinical use of the three different models is limited to seven years. As neither of the devices available today presents reliable clinical data on lifetime use, an assumption on expected lifetime is inevitable for the model to reflect the most probable scenario.

We fully acknowledge that the expected difference in the reimplantation rate is based on technical differences between the different solutions, and this is specified in Section 2.5.2 in Värendh et al. (2026) [6]. In our model, we chose to specifically target implant capabilities, as this is the main feature that would impact reimplantation (i.e., surgery). The statement that Sentio’s electromagnetic transducer is limited to 45 dB is incorrect; the Sentio implant is prepared for more power from an external sound processor. The fact that the external sound processor may come with different technical capabilities is not addressed in the model as this is not deemed to influence the risk of re-implantation. While we acknowledge how different capabilities of the sound processor to amplify sound could impact this, there are other features that would also steer benefits for patients. These include the bandwidth to which the amplification applies (e.g., Osia 400–7000 Hz vs. Sentio 200–9500 Hz) and how the amplified sound is processed and balanced to avoid feedback. For example, the maximum applicable gain is limited by feedback and is typically smaller than the technically measured maximum force output from a device (Dingemanse and Toll 2026 [16]). That is, the feedback management system will ultimately decide how well one can utilize the amplification. For Sentio and Ponto, Open Sound Optimizer will prevent feedback from occurring, and, as shown by Hol et al. (2026) [10], there are almost no reports of feedback issues in clinical use, indicating that amplification from Sentio is fully available to patients.

The main purpose of introducing planned reimplantation into the model was to reflect the literature (Caversaccio et al. 2025 [8]) and expected patient trajectories. As reported by Caversaccio et al., explantations and reimplantations do occur, and it would be unrealistic to remove these features completely. We acknowledge that the reimplantation feature introduces uncertainties, and several mitigations have been introduced to handle these; all are reported in the paper. Section 2.5.2 in Värendh et al. (2026) [6] describes how the introduction of a gamma distribution accounts for variability and how sensitivity analyses explore different time horizons (5, 10, 20, and lifelong), variations in timing, and exclusion of the parameter altogether:

*“To capture variability across patients and to avoid discrete jumps in costs and outcomes, both events were modelled using a gamma distribution.”*

(Section 2.5.2 in Värendh et al. (2026) [6])

*“Different time horizons (5, 10, 20, and lifelong) and variations in timing of anticipated reimplantation and SP replacement, excluding the parameter of anticipated reimplantation altogether, were explored as part of the sensitivity analyses.”*

(Section 2.5.2 in Värendh et al. (2026) [6])

More specifically, deterministic scenario analyses were added, in which all comparators were assumed to have equal reimplantation intervals of 10 years and 15 years. The results (Table A4 in Appendix B, Värendh et al. (2026) [6]) demonstrate that the overall conclusions regarding the cost-effectiveness of Sentio relative to Osia remain unchanged:

- Excluding anticipated reimplantation entirely: Cost savings fall by 61.8%; Sentio remains dominant.
- Setting anticipated reimplantation to 15 years for both Sentio and Osia: Cost savings fall by 69.5%; Sentio remains dominant.
- Setting anticipated reimplantation to 10 years for both Sentio and Osia (matching the IFU figure cited in the letter): Cost savings fall by 76.3%; Sentio remains dominant.

The 62% cost-differential figure cited in the letter is correct, and it is reported transparently in Table 6 and discussed in Section 4 in Värendh et al. (2026) [6]. It describes the cost component most sensitive to longevity assumptions, not a contingent driver of the cost-effectiveness conclusion: *“When anticipated reimplantation was excluded, Sentio remained dominant, although the magnitude of cost savings was reduced compared with the base case. This underscores the importance of accounting for reimplantation in long-term models, since excluding it risks underestimating lifetime costs.”* (Section 4 in Värendh et al. (2026) [6])

**Comment 5.** **Implications for Clinical and HTA Decision-making**

Cost-effectiveness analyses can inform decision-making, but their value depends entirely on methodological rigor and appropriate uncertainty handling. In the Värendh study, the estimated QALY gain derives entirely from differences in modeled complication rates based on disparate evidence quality, not from demonstrated superior outcomes. Moreover, the majority of long-term cost differences rely on unsubstantiated device longevity assumptions. For clinicians and other decision-makers considering BCHI options, this analysis does not provide reliable comparative-effectiveness evidence, and the conclusion of dominant cost-effectiveness should be viewed with considerable skepticism. Decisions should prioritize demonstrated patient outcomes, audiological performance data, and patient quality of life rather than a focus on economic models built on weak comparative evidence. Establishing dominance requires rigorous comparative data on both clinical outcomes and costs. The Värendh study falls short of this standard and should not influence clinical practice patterns or HTA decisions without substantial correction or additional evidence.

*Authors’* *response:*

We share the view that clinical and HTA decision-making should prioritize demonstrated patient outcomes, audiological performance, and quality of life. The conclusions of our analysis are framed accordingly throughout the manuscript:

*“The results demonstrate economic favourability rather than clinical superiority.”*

(Section 4 in Värendh et al. (2026) [6])

*“The magnitude of the incremental QALY gain was modest and likely within the measurement uncertainty of generic utility instruments… and therefore should not be interpreted as indicating clinically meaningful superiority.”*

(Section 4 in Värendh et al. (2026) [6])

The study contributes the first cost-effectiveness analysis of Sentio in the Australian healthcare setting, conducted in a context where HTA submissions for newly introduced devices are required despite the inherent asymmetry between the long-term evidence available for established comparators and the shorter-term evidence available for new entrants. This asymmetry is not unique to BCHIs; it arises routinely across medical devices, surgical interventions, and rare-disease therapies. To address it, our analysis has done the following:

First, the model follows established health-economic-modeling standards consistent with the ISPOR-SMDM Modelling Good Research Practices and the CHEERS 2022 reporting standard (Caro et al. 2012 [2]; Husereau et al. 2022 [4]) and is adapted from a peer-reviewed framework (Brunner et al. 2024 [5]), against which it was externally validated within 0.1% (Table A5 in Appendix C, Värendh et al. (2026) [6]).

Second, the parameters identified as most influential—*on-BCHI* and *off-BCHI* utility, reimplantation interval, and Sentio device price—were tested through extensive deterministic scenario analyses and a 10,000-iteration PSA. The results and their implications are reported in full in Section 3.2 and Table A4 and Table A5 in Värendh et al. (2026) [6], including the alternative-evidence substitutions proposed in the letter; across all of them, the economic favorability of Sentio relative to Osia is preserved.

Third, where direct comparative data are limited or absent, structured input from clinical experts was used to validate the model’s assumptions (Soares et al. 2024 [14]). These experts are senior ENT/audiology consultants with >15–25 years of experience treating patients with BCHIs across all relevant systems—i.e., the clinicians who in practice decide between Sentio, Osia, Ponto, and Baha Attract for individual patients in clinical use.

Fourth, the manuscript discusses the resulting uncertainty and its implications extensively in Section 4 in Värendh et al. (2026) [6], including the modest magnitude of the QALY gain, the dependence of dominance on specific modeling assumptions, and the priority of generating direct long-term Sentio data in future research.

On this basis, decision-makers involved in HTA processes have, in our view, sufficient transparent information to evaluate the comparative cost-effectiveness of Sentio alongside Osia, Ponto, and Baha Attract in the Australian setting. We trust that the journal’s reviewers considered these same elements—methodological standards, sensitivity analyses, expert validation, and transparent discussion of uncertainty—when accepting the manuscript for publication.

We thank the author for his engagement with our work and the journal editors for the opportunity to respond.

## Figures and Tables

**Table 1:** The full mapping of patient-reported and audiological outcomes across systems.

	**Active Transcutaneous**	**Osia**	**Sentio**
GBI	33 [23; 43]—Gutierrez et al. 2024 [12]	-	29 [24; 34]—Hol et al. 2026 [10]
SSQ total gain	Gutierrez et al. 2024 [12]	2.5—Briggs et al. 2022 [11]	2.8—Hol et al. 2026 [10]
- Speech gain	- 2.9	- 2.7	- 2.9
	- 95%CI [2.0; 3.9]		- 95%CI [2.4; 3.3] *
- Spatial gain	- 2.9	- 2.3	- 2.7
	- 95%CI [1.6; 2.8]		- 95%CI [2.1; 3.3] *
- Quality of hearing gain	- 2.5	- 2.4	- 2.6
	- 95%CI [1.7; 3.3]		- 95%CI [2.2; 3.1] *
Functional gain	-	35 [29; 41] Key et al. 2024 [13]	33 [30; 36] Hol et al. 2026 [10]

Data are presented as means, with 95% confidence intervals when available. * Data on file Oticon Medical AB.

## Data Availability

All data were extracted from published sources. Formulas extracted from public sources will be available on request, directed to M.V.
